# Waist Circumference Cutoff Points to Predict Obesity, Metabolic Syndrome, and Cardiovascular Risk in Turkish Adults

**DOI:** 10.1155/2013/767202

**Published:** 2013-11-27

**Authors:** Alper Sonmez, Fahri Bayram, Cem Barcin, Muge Ozsan, Ahmet Kaya, Vedia Gedik

**Affiliations:** ^1^Department of Endocrinology and Metabolism, School of Medicine, Gulhane Military Medical Academy, 06018 Etlik Ankara, Turkey; ^2^Department of Endocrinology and Metabolism, School of Medicine, Erciyes University, Talas, 38039 Kayseri, Turkey; ^3^Department of Cardiology, School of Medicine, Gulhane Military Medical Academy, 06018 Etlik Ankara, Turkey; ^4^Department of Endocrinology and Metabolism, Nigde State Hospital, 51100 Nigde, Turkey; ^5^Department of Endocrinology and Metabolism, School of Medicine, Necmettin Erbakan University, Meram, 42080 Konya, Turkey; ^6^Department of Endocrinology and Metabolism, School of Medicine, Ankara University, Sıhhıye, 060100 Ankara, Turkey

## Abstract

*Objective*. The waist circumference (WC) cutoff levels defined for the Caucasian people may not be representative for different ethnic groups. We determined sex specific WC cutoff points to predict obesity, metabolic syndrome, and cardiovascular risk in Turkish adults. *Design and Methods*. The demographic characteristics of 1898 adult males and 2308 nonpregnant females from 24 provinces of 7 different regions of Turkey (mean age 47 ± 14 yrs) were evaluated. *Results*. The WC levels of 90 cm and 100 cm define overweight and obese males while the levels of 80 cm and 90 cm define overweight and obese females. With these cutoff values, 239 additional males (12.6%) are diagnosed as overweight and 148 additional males (7.8%) as obese. Instead, 120 females (5.1%) are free of being labeled as obese. *Conclusions*. This is the first nationwide study to show the action levels of WC for overweight and obese Turkish adults. The ideal cutoff levels of WC to predict metabolic syndrome are 90 cm and 80 cm for Turkish adult men and women, respectively. These values are easy to implement and suggested to be used by the physicians dealing with cardiometabolic disorders in Turkey.

## 1. Introduction

Obesity is a global epidemic affecting more than one third of the adult population in Turkey [1, 2] as in the rest of the world [3–5]. Although the rise in obesity prevalence brings about a significantly increased mortality risk [6, 7], it is not practical to manage all the obese subjects with a global program. Therefore, subjects with the significantly increased risk of morbidity and mortality should be identified and prioritized for any intervention. For this, an appropriate and accurate measure of obesity should be at hand.

The body mass index (BMI) is a convenient and widespread measure of obesity. However, it is just a crude proportion, which does not take into account the amount of abdominal fat mass [8]. Waist circumference (WC), on the other hand, is an easy and reliable measure of visceral adipose tissue and a simple index of cardiovascular risk [9, 10]. The World Health Organization (WHO) reported sex specific WC cutoff values for the Caucasian people, named as action levels 1 and 2, to define the overweight or obese people [8]. These cutoff values, originally established in a Dutch population [11, 12], were later adopted by several medical organizations in order to define Metabolic Syndrome [13, 14]. However, these cutoff levels may not necessarily represent the characteristics of the other populations. Therefore, it is recommended that the sex specific WC cutoff points should be established for different ethnic groups [8, 13].

Many reports have been published so far to describe the WC cutoff points of different populations. However, most of these reports show discrepant results even within the same ethnic groups [15, 16]. The main reason of the discrepancies is the use of different methodologies to establish the WC cutoff points. Likewise, different reports show different WC cutoff points in Turkish adult men and women [17–20]. Also, the WC cutoff values do not define overweight and obesity in these studies by using the criteria in the reference studies [11, 12] adored by WHO [8]. Therefore, we aimed to investigate whether the suggested sex specific WC cutoff points for the Caucasian adults are appropriate for the Turkish adult population. The secondary aim of the study is to search for better WC cutoff points to predict obesity, metabolic syndrome, and increased cardiovascular risk in Turkish adult men and women.

## 2. Methods and Procedures

### 2.1. Study Population and Sampling

This cross-sectional study was conducted by the *Obesity Hypertension and Lipid Study Group of the Turkish Society of Endocrinology and Metabolism *in 24 provinces from the 7 regions of Turkey. The detailed description of the study protocol is given elsewhere [1]. The multistage probability sampling design was used to select the cases. In brief, Turkish adult males and nonpregnant females were randomly enrolled from both the provincial district centers and villages, considering the demographics, economic, social, and geographical statuses. Approval was obtained from the Ethical Committee of the Ministry of Health and the household identification form (HIF) data were obtained from the Primary Health Care Centers of the Provincial Health Directorates affiliated to the Ministry of Health. The study was employed in accordance with the Declaration of Helsinki. The informed consents were obtained from all participants, before the enrolment.

At least 3 provinces were selected from each region by a random sampling method. The populations of these 7 regions were obtained from the records of the 2000 census. The study sample included males and nonpregnant females aged between 20 and 83 years. The populations of city centers, districts, and villages were classified by using the stratified sampling method and then were selected from the HIF data by a random sampling method. The geography of Turkey was classified into three groups according to altitude. Sea level was accepted as zero. 0–300 m was taken as coastal, 300–900 m as moderate elevation and 900 m and above as high elevation.

The medical histories and demographical data of the participants were obtained and structured questionnaires were completed with face-to-face interviews. The personal and family histories of hypertension, diabetes mellitus, cardiovascular diseases, and other chronic diseases were obtained. Heights and weights of participants were measured. When the subjects were weighed, they were asked to take off their shoes and any other belongings that could possibly add extra weight. BMI was calculated by dividing the weight (in kg) by the height in meters squared. WC of the participants were measured at the level of the iliac processes and the umbilicus with a soft tape measure to evaluate abdominal obesity. The same definitions for overweight and obese people as in the original Dutch study [8] were used. Accordingly, the participants with either the BMI ≥ 25 kg/m^2^ or BMI < 25 kg/m^2^ but WHR > 0.95 in men or >0.80 in women were defined as overweight. Subjects with the BMI ≥ 30 kg/m^2^ in both sexes were considered obese. Systolic blood pressure (SBP) and diastolic blood pressure (DBP) were measured twice in the sitting position, with an interval of 15 min between the measurements, after a rest period for 30 min. The mean of these two measurements obtained by a standard sphygmomanometer was recorded as final value of blood pressure. The participants whose SBP ≥ 130 mmHg and/or DBP ≥ 90 mmHg as well as the ones who were on antihypertensive medications were diagnosed to have hypertension. Metabolic syndrome was diagnosed as the presence of at least 3 of the following parameters, according to Adult Treatment Panel III criteria: abdominal obesity (WC > 102 cm for males and >88 cm for females), hypertension (SBP > 130 mmHg and/or DBP > 85 mmHg) or history of antihypertensive usage, hypertriglyceridemia (≥150 mg/dL) or presence of treatment for this disorder, low HDL-C (<40 mg/dL in males and <50 mg/dL in females), and high fasting plasma glucose (≥100 mg/dL) or presence of diagnosis of type 2 diabetes [14].

The venous blood samples were taken between 7:00 and 10:00 a.m after an overnight fasting. Blood samples were centrifuged at room temperature for 10 min at 3000 rpm and the sera were stored in ice bags and placed into deep freezers at −70°C on the same day. Glucose, total cholesterol, high-density lipoprotein cholesterol (HDL-C) and triglyceride (TG) levels were measured by the enzymatic spectrophotometric method with the Kone Lab Auto Analyzer (Thermo Clinical Labsystems Oy Vantaa, Finland). Low-density lipoprotein cholesterol (LDL-C) was calculated by the Friedewald formula (in those with a triglyceride level of below 400 mg/dL). Diabetes mellitus was diagnosed according to the American Diabetes Association (ADA) criteria. Accordingly, single fasting blood glucose of above 126 mg/dL was considered to be the evidence of diabetes mellitus. Those with a previous diagnosis of diabetes mellitus and using oral antidiabetics and/or insulin were also considered as diabetics. Impaired fasting glucose was defined as fasting blood glucose levels between 100 and 126 mg/dL.

#### 2.1.1. Statistical Analysis

Continuous variables were shown as mean ± SD. and categorical variables as percentage. Student's *t*-test was used to compare continuous variables.

Action level 1 and 2 were calculated as WC values to predict overweight and obese individuals, respectively. Receiver operating curve analysis was used to calculate sensitivity and specificity. The level of WC where the sum of the sensitivity and specificity is the highest was considered as the cutoff point.

Positive prediction for the Metabolic Syndrome was calculated as the percentage of subjects with a WC above the action level who have two or more risk factors other than WC criterion (considering the individual would have at least 3 of 5 metabolic syndrome criteria after adding the WC). Negative prediction for the Metabolic Syndrome was calculated as the percentage of subjects with a WC below the action level and with two or less Metabolic syndrome risk factors.

All statistical analyses were calculated using SPSS version 15.0 (Chicago, IL). Two-tailed *P*-values of <0.05 were considered to be statistically significant.

## 3. Results

**Figure 1:**
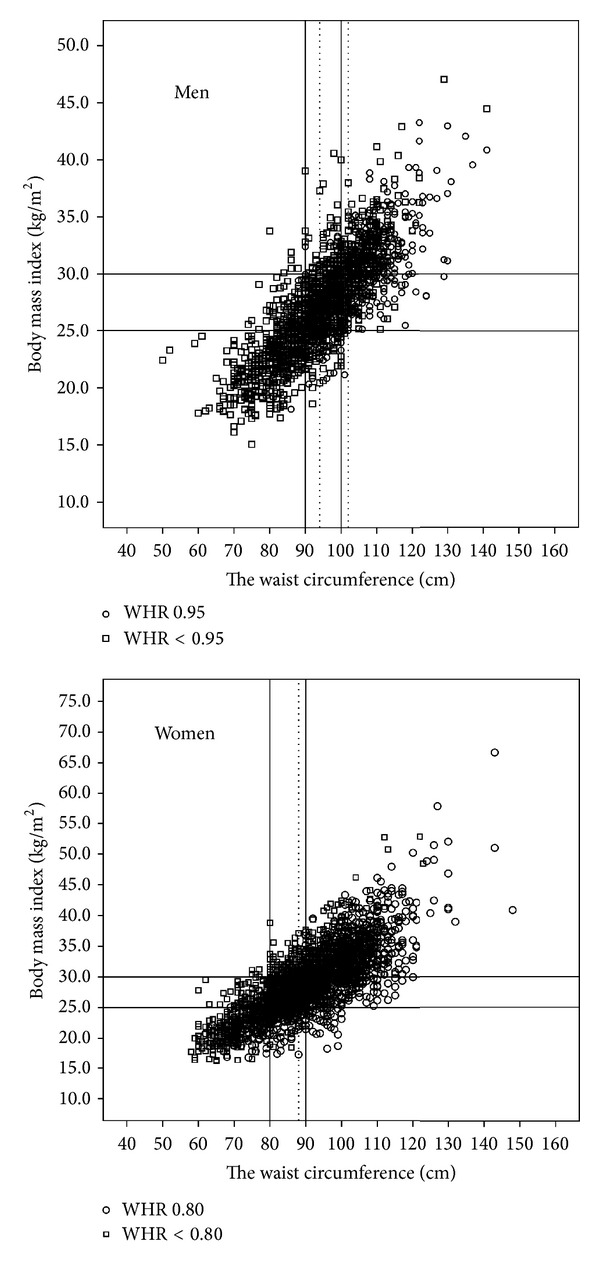
The relationship of waist circumference (WC) and body mass index in Turkish adults and cutoff values of WC both recommended by World Health Organization and calculated in the present study. The relation between WC levels and BMI in men and women and the two action levels of WC that identify subjects with BMI ≥ 25 kg/m^2^ or ≥30 kg/m^2^ and with WHR ≥ 0.95 for men and ≥0.80 for women. Dotted lines show action levels 1 and 2 recommended by the WHO. Solid lines show the action levels determined by the present study. ∘ indicates the individuals with WHR ≥ 0.95 (in men) and ≥0.80 (in women); false negative in upper left quadrant. □ indicates the individuals with WHR < 0.95 for men and <0.80 for women; false positive in lower right quadrant. Linear Regression: BMI = (WC × 0.297) − 0.975 in men; BMI = (WC × 0.338) − 1.32 in women.

The demographic characteristics of the study population are given in Table 1. According to the data, 37.3% of the adult population is overweight and 36% is obese. The prevalence of obesity in Turkish women (42.5%) was significantly higher than that of the Turkish men (28.0%) (*P* < 0.001). On the other hand, the male population had significantly higher number of smokers, higher serum triglyceride and total cholesterol, and lower HDL cholesterol levels (*P* < 0.001 for all). The plot graphs giving the distribution of the WC levels for females and males related to BMI divided by the proposed action levels are given in Figure 1.

**Figure 2:**
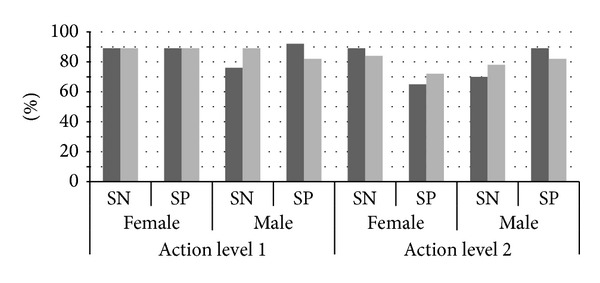
The sensitivity and specificity of the waist circumference cutoff levels recommended by the WHO and the levels calculated from the Turkish population. SN: sensitivity, SP: specificity, bold bars: the criteria of the WHO, The light Bars: The Criteria measured for the Turkish adult population.

The sensitivity and specificity of the WC cutoff levels recommended by the WHO and the levels calculated from the Turkish population in the present study are given in Figure 2. The cutoff level for the action level 1 to predict the overweight men is calculated as 90 cm, and the cutoff level for the action level 2 to predict the obese men is calculated as 100 cm. These cutoff values for the Turkish males, are lower than those proposed by the WHO (94 cm and 102 cm resp.) and have higher sensitivities and lower specificities. On the other hand, the cutoff level for the action level 1 to predict the overweight women is the same with the level proposed by the WHO (80 cm). But, the cutoff level for the action level 2 to predict obese Turkish women (90 cm) is higher than that of the level of the WHO (88 cm). Consequently, the action level 2 to detect obese Turkish women has decreased sensitivity and increased specificity when compared to the level proposed by the WHO (Figure 2).

The positive and negative predictive values of these cutoff values for the diagnosis of metabolic syndrome are mentioned in Table 2. These cutoff levels are also calculated for each of the components of metabolic syndrome and are given in Table 2.

The 10-year Framingham cardiovascular risk ratios of the participants categorized according to different WC cutoff values are given in Table 3. The 10-year cardiovascular risk ratios are significantly different in each category, either calculated according to the WHO criteria or according to the proposed cutoff levels in this study. When the WC levels of 90 cm and 100 cm are used instead of 94 cm and 102 cm for the diagnosis of action levels of the male population, 239 additional subjects (12.6%) are detected as overweight and 148 additional subjects (7.8%) are diagnosed as obese. Moreover, when the WC level of 90 cm is used instead of 88 cm for the diagnosis of action level 2 in the female population, 120 subjects (5.1%) can be free of being labeled as obese.

## 4. Discussion

The WC cutoff levels calculated in this study are not the same but rather close to those levels recommended for the Caucasian adults [8] or the values established in the previous reports for the Turkish adult population [17–20]. The cutoff levels, which we recommend according to the present study, are practical and have several advantages on the previously recommended values. By reducing the previously defined action levels of ≥94 cm and ≥102 cm to ≥90 cm and ≥100 cm for the Turkish adult men, 12.6% more overweight subjects and 7.8% more obese subjects can be detected. Again, by taking the action level 2 as 90 cm instead of 88 cm defined for the Caucasian women, 5.1% of Turkish women can be free of being labeled as obese. The results also show that the cutoff values of 80 cm for the females and 90 cm for the males are appropriate for the diagnosis of Metabolic Syndrome in this specific population. The reasons for the discrepancies from the previous reports and the clinical implications of the present findings are addressed below.

Visceral adipose tissue is no longer regarded as a depot but an active endocrine organ coordinating a variety of biological processes including energy metabolism, neuroendocrine, and immune functions [21]. WC is an easy and reliable surrogate marker of the visceral adipose tissue mass and a simple index of cardiovascular risk [9, 10]. Visceral obesity, determined by increased WC, is a significant risk for the cardiovascular morbidity and mortality and is one of the diagnostic criteria of the Metabolic Syndrome [13]. However, it is well reported that distinct ethnic groups may have significantly different visceral adipose tissue distributions and different cardiometabolic risk profiles [16, 22, 23]. Therefore, the identification of risk by using WC is population specific and depends on levels of obesity and other risk factors for cardiovascular disease and type 2 diabetes mellitus [8]. The WC cut points generally recommended for the Caucasian adults [8, 13] are inferred from a Dutch population [11, 12]. The demographic characteristics of the Dutch people are not likely to represent those of the Turkish adults. Therefore, the WC cutoff values, established in this study, should be used to better estimate the presence of metabolic syndrome and the risk of cardiovascular diseases.

Many research papers have been published so far in order to report specific WC cutoff points in different populations. However, the results of these studies significantly differ, even within the same ethnic groups [15, 16]. Using different methods to measure WC and taking distinct health outcome measures to establish cutoff levels are among the reasons of the discrepancies between the studies. The widely accepted sex specific WC cutoff values for the Caucasian adults were initially adopted by the WHO [8]. These cutoff values were originally calculated in a Dutch population to establish the overweight (BMI > 25 kg/m^2^ or WHR > 0.95 in men or >0.80 in women) and obese (BMI > 30 kg/m^2^ or WHR > 0.95 in men or >0.80 in women) adults [11]. Given the names of *“action level 1”* (means no further weight gain) and* “action level 2”* (*should reduce weight*), these cutoff values point out significant increases in the cardiovascular event risk [12]. The parameters to define the increased cardiovascular risk in the Dutch study were high blood pressure, high levels of total cholesterol and low levels of HDL cholesterol [12]. However, different outcome measures were used to calculate the cutoff values in the subsequent studies, including hyperglycemia (either impaired fasting glucose, impaired glucose tolerance, or diabetes mellitus), high LDL cholesterol, Triglycerides, Metabolic syndrome, Coronary heart disease, cardiovascular disease, and overall mortality [15]. Using different outcome measures in calculation, for sure causes different results for the cutoff values. Another significant reason for the discrepant cutoff values is the use of different methods of optimizing sensitivity and specificity [15]. Published studies have either taken the WC where the sum of the sensitivity and the specificity is the highest or the point where the sensitivity and the specificity are nearly equal. Some authors on the other hand, have used the point on the ROC curve where the distance to the upper left corner is the shortest. Of note, the method regarding to this issue was not given in some studies. In our study we have chosen the cutoff values, where the sum of sensitivity and specificity is the highest. This detection limit allowed us to cover an optimal number of overweight or obese adults when compared to the current criteria recommended for the Caucasian adults.

Few studies reported sex specific WC cutoff levels of the Turkish adults [17–20]. All these studies used different methods to establish the cutoff limits. A study reported the WC cutoff levels for the prediction of the increased insulin resistance, as 93 cm for men and 83 cm for women. These thresholds, however, do not label any specific BMI limit or any significant increase in the cardiovascular risk [19] Onat et al. mentioned distinct WC action levels as 87 cm and 95 cm for the Turkish men [17] and 83 cm and 91 cm for the Turkish women [18]. These action levels do not show any specific BMI or WHR limits, but point out to increased risk of dyslipidemia, hypertension and Metabolic Syndrome in action 1 and the increased risk of coronary heart disease and type 2 diabetes in action 2. It is for sure, important to establish cutoff levels to predict the increased risk of metabolic disorders and coronary heart diseases. However, in order to compare the demographic properties and the cardiometabolic risk states of the different populations, the action levels should be defined by using similar criteria. Therefore, the cutoff levels in our study were calculated by using the methods of the original studies [11, 12]. Namely, the action level 1 in our study defines the point of BMI > 25 kg/m^2^ or WHR > 0.95 in men or >0.80 in women. The action level 2 on the other hand defines the BMI > 30 kg/m^2^. Although one study has used the above criteria to establish WC cutoff limits so far, this study was performed in an obesity outpatient clinic of a university hospital and reported only the WC cutoff points for women in Turkey [20]. Still and interestingly, the action levels 1 and 2 for the women participated in this study were 81 cm and 90 cm, respectively, very similar to our results.

The WC cutoff levels, given in the present study, are appropriate to identify the overweight and obese Turkish adults and have reasonable power to establish people with increased cardiometabolic risk. The Framingham risk ratios of the subjects below the action level 1 significantly disperses from those above the action level 2. Moreover, the high negative predictive values for the diagnosis of metabolic syndrome indicate that it is very unlikely to have metabolic syndrome if the WC of a Turkish adult is below the action level 1. Therefore, it would be wiser to take the cutoff values 90 cm and 80 cm for action level 1 for the diagnosis of Metabolic Syndrome in Turkish men and women. Although calculated by different methods, these cutoff values are somewhat close to the previously reported cut points for the Turkish men [17, 19] and women [18–20]. Using the action levels given in our study has two significant advantages. Firstly these levels reflect the cutoff values of the Turkish adult population, calculated by the generally recognized methods. Secondly, these cutoff levels are very easy to recall and implement which would be practical for the family physicians and internists dealing with obesity.

This study however has several limitations. Due to the cross sectional design, the action levels only give us the risk estimates, not the outcomes. Therefore, using the cutoff points inferred from the outcome studies may still be advantageous. Another limitation may be the substantially low 10-year cardiovascular event risk calculated according to Framingham risk score. Even the subjects having WC above the action level 2 have moderate calculated cardiovascular risk. Because the Turkish population is regarded to have high cardiovascular risk, the validity of the Framingham risk score to predict hard clinical end points in Turkish population may be questioned.

In conclusion, this is the first nationwide study to show the action levels of WC to predict overweight and obese Turkish adults using the same criteria of the original Dutch study. These levels, however, are not very much different from the previous values recommended for the Caucasian men and women. The WC cutoff levels to diagnose metabolic syndrome can be established as 90 cm and 80 cm for Turkish adult men and women respectively. Finally, it would be pragmatic for the physicians to accept these cutoff values in Turkey, and in areas with large Turkish immigrant populations.

## Figures and Tables

**Table 1:** The demographic characteristics of the Turkish adult population.

	General population (*n* = 4206)	Males (*n* = 1898)	Females (*n* = 2308)	*P*
Age [years]	47.0 ± 14.8	48.9 ± 14.6	45.3 ± 14.7	**<0.001**
SBP [mm Hg]	133.1 ± 26.4	132.5 ± 25.6	133.5 ± 27.1	0.24
DBP [mm Hg]	81.2 ± 14.8	81.0 ± 15.0	81.4 ± 14.7	0.44
BMI [kg/m^2^]	28.4 ± 5.2	27.4 ± 4.4	29.1 ± 5.7	**<0.001**
WC [cm]	92.6 ± 12.8	95.5 ± 11.8	90.3 ± 13.1	**<0.001**
WHR	0.87 ± 0.9	0.91 ± 0.07	0.83 ± 0.08	**<0.001**
Fasting BG [mg/dL]	103.1 ± 45.2	102.8 ± 42.0	104.7 ± 47.6	0.19
Triglycerides [mg/dL]	145.2 ± 96.9	152.6 ± 104.0	139.2 ± 90.1	**<0.001**
Total chol. [mg/dL]	194.7 ± 47.5	191.7 ± 47.3	197.1 ± 47.6	**<0.001**
HDL-C [mg/dL]	49.7 ± 15.3	46.2 ± 14.4	52.6 ± 15.4	**<0.001**
LDL-C [mg/dL]	117.7 ± 41.0	117.1 ± 40.7	118.1 ± 41.2	0.45
Smoking [*n* (%)]	1024 (24.3)	749 (39.5)	275 (11.9)	<0.001*
Overweight [*n* (%)]	1569 (37.3)	797 (42.0)	772 (33.4)	**<0.001**
Obesity [*n* (%)]	1513 (36.0)	531 (28.0)	982 (42.5)	**<0.001**

The comparisons were done by independent samples *t* test. *Chi-Square test. SBP: Systolic blood pressure, DBP: Diastolic blood pressure, BMI: Body mass index, WC: Waist circumference, WHR: Waist to hip ratio, fasting BG: Fasting blood glucose. The results are given as mean ± SD.

**Table 2:** The positive and negative predictive values of different waist circumference cutoff points for metabolic syndrome and its individual components.

	Males								Females					
	Action Level 1				Action Level 2				Action level 1*		Action level 2			
WC cutoff levels (cm)	WHO (94)		Turkish (90)		WHO (102)		Turkish (98)		WHO/Turkish (80)		WHO (88)		Turkish (90)	
Predictive values (%)	*P*	*N*	*P*	*N*	*P*	*N*	*P*	*N*	*P*	*N*	*P*	*N*	*P*	*N*
Metabolic syndrome	64	87	62	91	70	82	69	84	62	92	67	86	69	85
Hyperglycemia	39	72	38	74	44	70	43	71	37	82	39	76	40	76
Hypertriglyceridemia	46	73	44	77	49	67	47	68	38	85	42	80	43	79
Low HDL	39	67	39	69	40	65	40	66	49	62	51	59	52	60
Hypertension	68	53	67	60	73	47	71	48	70	68	74	56	75	54

*P*: Positive predictive value, *N*: Negative predictive value.

*The cut off levels for the action level 1 to predict the overweight women according to the WHO or Turkish criteria are the same (80 cm).

**Table 3:** The 10-year Framingham cardiovascular risk ratios of the Turkish adults, calculated according to the different waist circumference categories using the thresholds proposed by the world health organization and the data of the present study.

	The values for the male population (*n* = 1898)				The values for the female population (*n* = 2308)			
	Cutoff (WHO)	10 years risk ratio	Cutoff (Turkish)	10 years risk ratio	Cutoff (WHO)	10 years risk ratio	Cutoff (Turkish)	10 years risk ratio
Normal	≤94 cm (*n* = 783) (41.8%)	9.0 ± 9.3	≤90 cm (*n* = 544) (28.7%)	7.6 ± 8.2	≤80 cm (*n* = 465) (20.1%)	2.5 ± 3.7	≤80 cm (*n* = 465) (20.1%)	2.5 ± 3.7

Overweight	94–102 cm (*n* = 551) (29.0%)	12.8 ± 11.4	90–100 cm (*n* = 642) (33.8%)	12.1 ± 10.7	80–88 cm (*n* = 470) (20.3%)	5.2 ± 6.5	80–90 cm (*n* = 590) (25.6%)	5.4 ± 6.4

Obese	≥102 cm (*n* = 564) (29.7%)	15.1 ± 12.2	≥100 cm (*n* = 712) (37.5%)	15.1 ± 12.4	88 cm (*n* = 1373) (59.5%)	8.9 ± 8.3	≥90 cm (*n* = 1253) (54.3%)	9.1 ± 8.5

P*		<0.001		<0.001		<0.001		<0.001

*The comparisons of the 10 yrs risk ratios in each category, performed by the ANOVA.
